# Supplementary material for: Subgenome Discrimination in Brassica and Raphanus Allopolyploids Using Microsatellites
Source: Cells. 2021 Sep 8;10(9):2358. doi: 10.3390/cells10092358 (PMC8466703; doi:10.3390/cells10092358)
Supplement: Supplementary file 1 [file cells-10-02358-s001.zip › cells-1335960-supplementary.pdf]

cells

Supplementary Materials

Table S1. Summary of PLOPs used for FISH with their corresponding length (base), fluorochrome, and notes on the detection of FISH signals in the corresponding genomes.

New Name	PLOP	Length	Fluorochrome	Note[a]
ACBR_msat01	AGAGAGAGAGAGAGAGAGAGAG	22	Cy3-5′	B/R[b]
ACBR_msat02	ATATATATATATATATATATAT	22	TxR-5′	C/R
ACBR_msat03	AAGAAGAAGAAGAAGAAGAAG	21	FAM-5′	A/C/B
ACBR_msat04	GATGATGATGATGATGATGAT	21	Cy5-5′	ND[b]
ACBR_msat05	ATTATTATTATTATTATTATT	21	FAM-5′	ND
ACBR_msat06	TTTAGGGTTTAGGGTTTAGGGTTTAGGG	28	Coumarine-5′	A/C/B/R
ACBR_msat07	AACAACAACAACAACAACAAC	21	FAM-5′	ND
ACBR_msat08	GTGTGTGTGTGTGTGTGTGTGT	22	Cy3-5′	A/C/B/R
ACBR_msat09	CCTCCTCCTCCTCCTCCTCCT	21	Cy3-5′	A/C/B/R
ACBR_msat10	AAATAAATAAATAAATAAAT	20	FAM-5′	ND
ACBR_msat11	ACCACCACCACCACCACCACC	21	Cy3-5′	A/C/B/R
ACBR_msat12	AAAGAAAGAAAGAAAGAAAGAAAG	24	FAM-5′	ND
ACBR_msat13	CAGCAGCAGCAGCAGCAGCAG	21	TxR-5′	A/C/B/R
ACBR_msat14	TTTAGGGTTAGGTAGGGTTTAGGGTTA	27	Cy5-5′	A
ACBR_msat15	ACAAACAAACAAACAAACAAACAA	24	TxR-5′	ND
ACBR_msat16	TTCGGTTCGGTTCGGTTCGGTTCGG	25	TxR-5′	ND
ACBR_msat17	TAGTAGTAGTAGTAGTAGTAG	21	FAM-5′	ND
ACBR_msat18	ATAGATAGATAGATAGATAGATAGATA	27	Cy5-5′	ND
ACBR_msat19	TATTTTATTTTATTTTATTTTATTT	25	TxR-5′	C/R
ACBR_msat20	GGGTTTCGGGTTTCGGGTTTCGGGTTT	27	Cy5-5′	C
ACBR_msat21	AATTAATTAATTAATTAATTAATT	24	TxR-5′	ND
ACBR_msat22	ACTGTTCATCGAGATACTGTTCATCGA	27	FAM-5′	ND

[a] A, C, B, and R represent the clustered FISH signals detected in the corresponding genomes. Most FISH signals were non-clustered. [b] Showed signal in one homologous chromosome in the A genome. [c] ND: non-detected

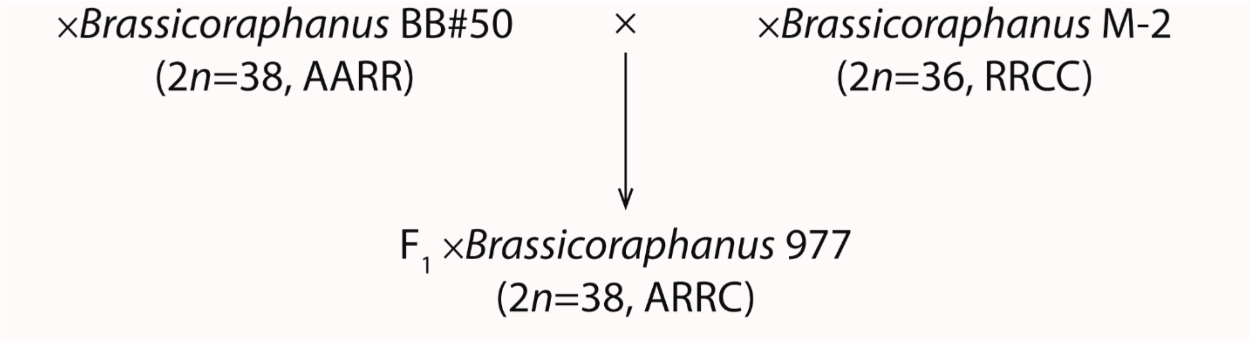

Figure S1. The hybrid ×*Brassicoraphanus* 977 that possible carries chromosomes blocks form A, C, and R genomes was developed from crosses of synthetic allotetraploids.

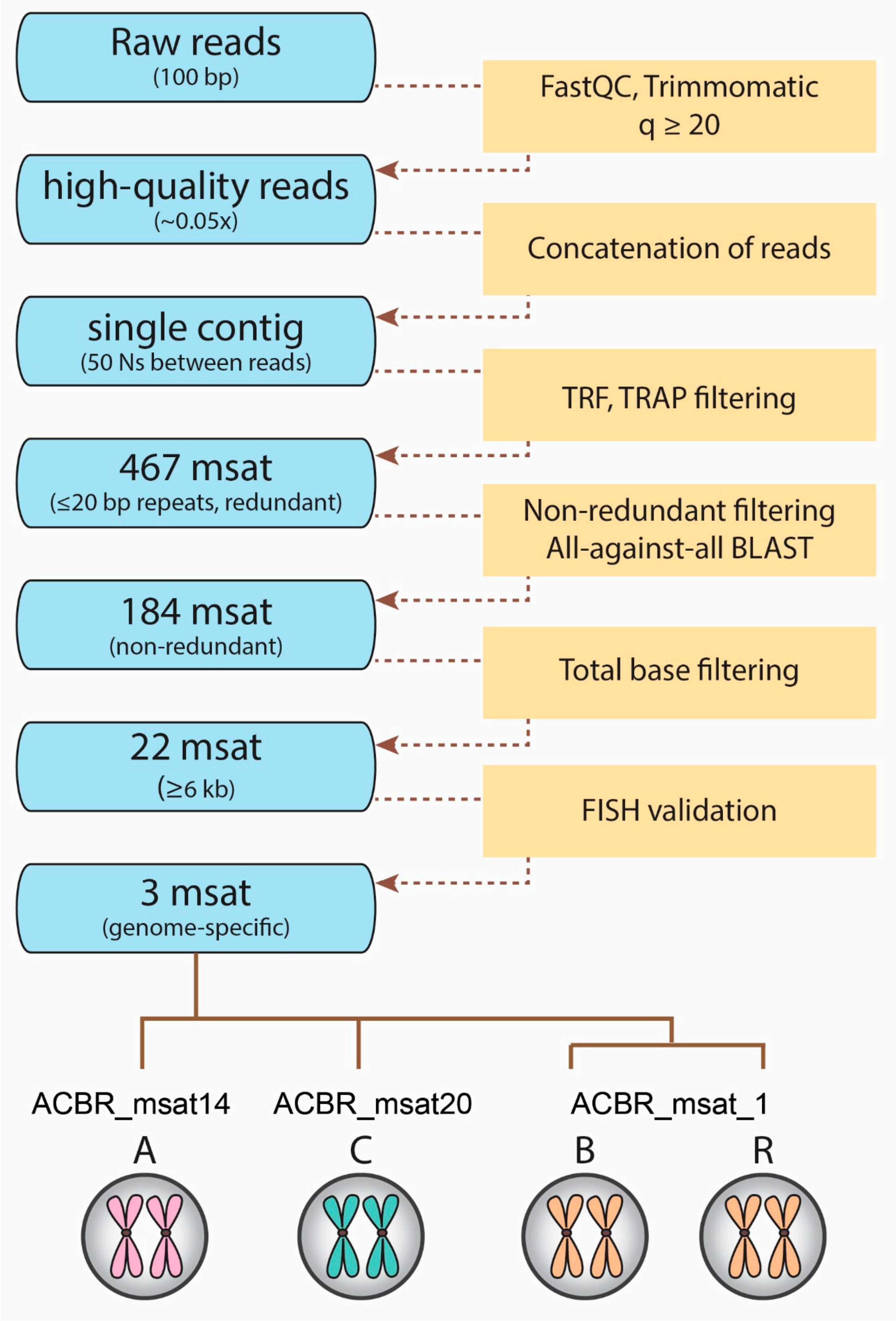

Figure S2. Summary of the microsatellite mining and FISH validation pipeline.

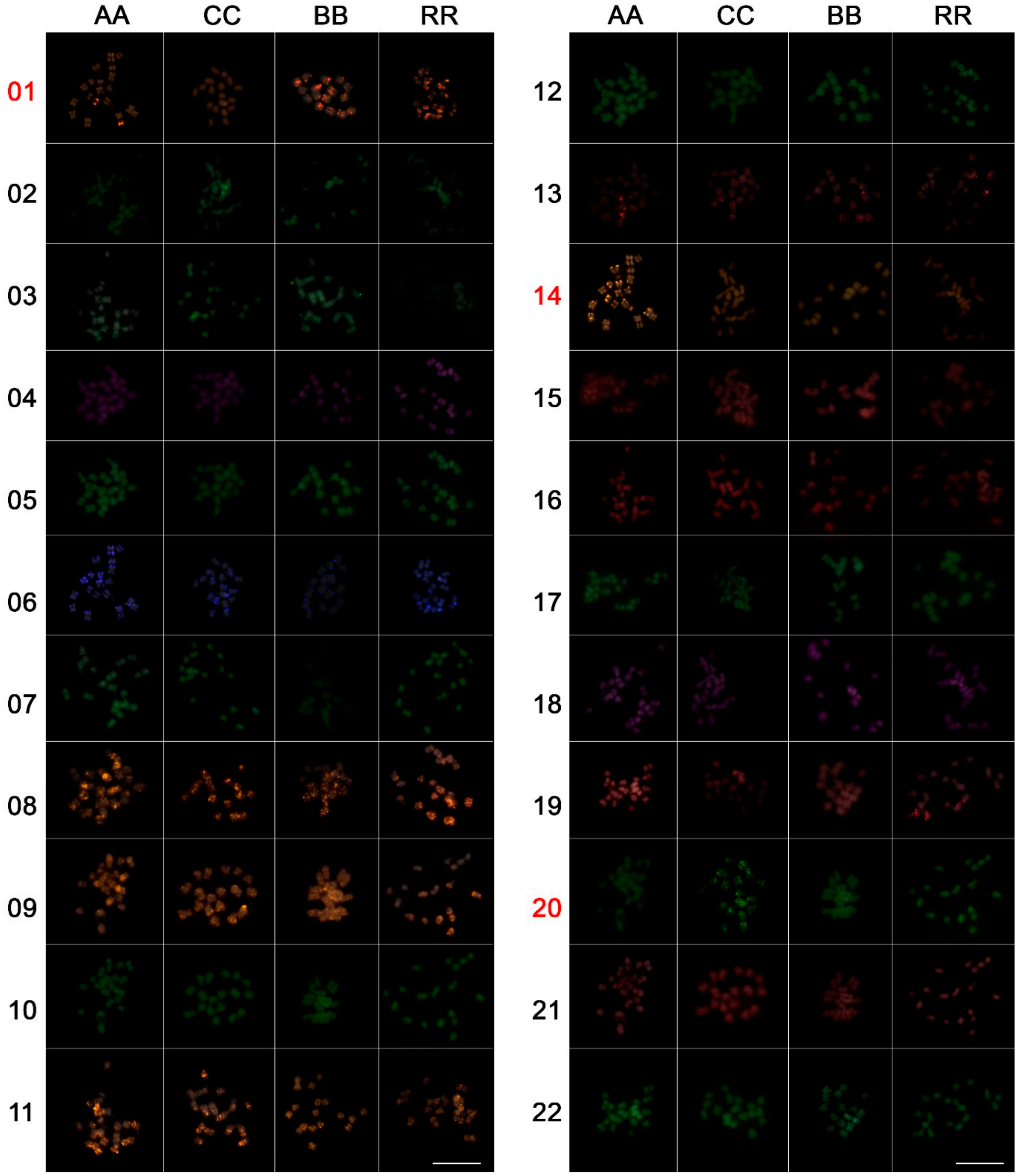

Figure S3. FISH screening of the 22 candidate microsatellites in the diploid *B. rapa* (AA), *B. oleracea* (CC), *B. nigra* (BB), and *R. sativus* (RR) genomes. While most FISH signals showed non-clustered loci, some showed clustered signals. The three microsatellite probes that were specific to the A, C, and B genomes in the U's triangle are highlighted red. Numbers correspond to the list of microsatellites in Table 3.

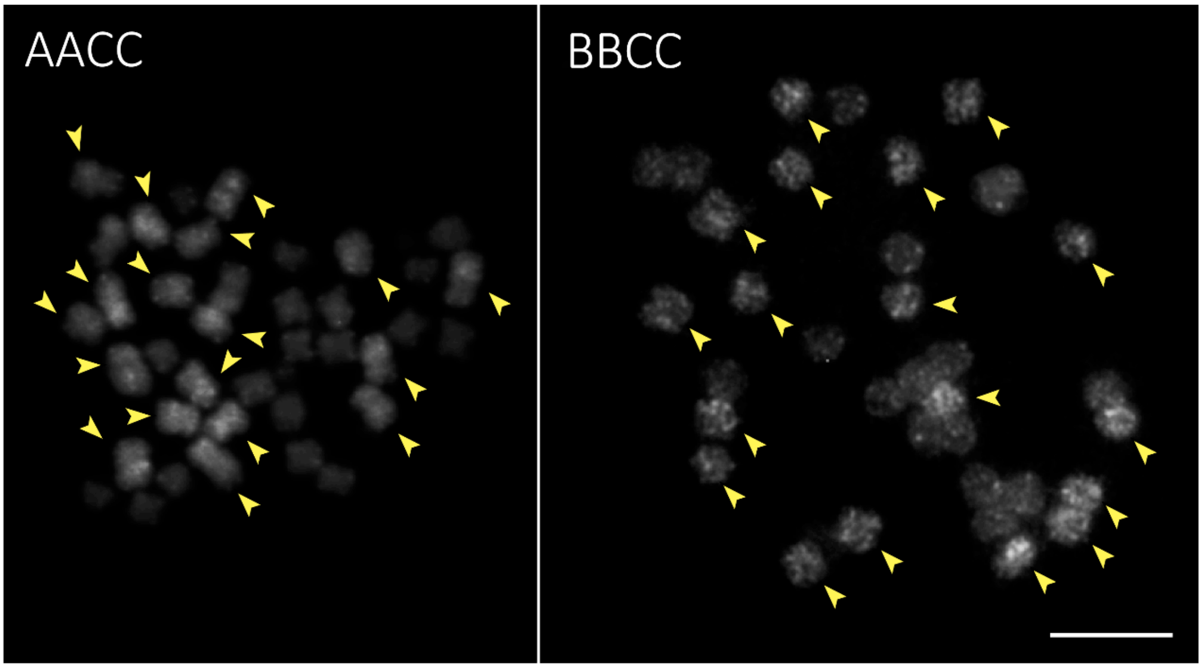

Figure S4. Chromosomal distribution of *Arabidopsis*-type telomere repeats (ACBR_msat06) showing more intense FISH signals in 18 C genome chromosomes (yellow arrowheads) in *B. napus* (AACC) and *B. carinata* (BBCC). This result shows the practicality of telomere repeat in discriminating C-genome chromosomes within allopolyploids with C- genome component. Scale bar = 10 μm.
